# Tobacco dependence among people with mental illness: a facility-based cross sectional study from Southwest Ethiopia

**DOI:** 10.1186/s13104-017-2608-7

**Published:** 2017-07-17

**Authors:** Zemenay Molla, Lamesa Dube, Wolfgang Krahl, Matiwos Soboka

**Affiliations:** 1 Bahir Dar Tseda Health Science College, BahirDar, Ethiopia; 2 Department of Psychiatry, College of Public Health and Medical Sciences, Jimma University, Jimma, Ethiopia; 3 Department of Epidemiology, College of Public Health and Medical Sciences, Jimma University, Jimma, Ethiopia; 4 Department of Forensic Psychiatry, Isar Amper Klinikum, Munich, Germany; 5 Center for International Health, Ludwig Maxmillians University, Munich, Germany

**Keywords:** Tobacco dependence, Khat use, Alcohol use disorders, Mental disorder, Sub-Saharan Africa, Ethiopia

## Abstract

**Background:**

Tobacco smoking is a health care issue in developed as well as in developing countries. Tobacco smoking among people with mental illness is significantly higher than in the general population. Tobacco smoking has negative effects on physical, mental and financial well-being of people with mental illness but little is known about tobacco dependence among mental health service users in sub-Saharan African countries, including Ethiopia. Therefore, this study attempted to assess the prevalence of tobacco dependence and associated factors among mental health service users at Jimma University teaching hospital.

**Methods:**

A cross-sectional study was conducted among 305 male and 117 female mental health service users at Jimma University teaching Hospital in 2014. The Fagerstrom Test for Nicotine Dependence (FTND) was used to assess tobacco dependence. Logistic regression analysis was used for bivariate and multivariate analysis. Variables with a P value of <0.05 were considered to be associated with tobacco dependence in the final model.

**Results:**

The prevalence of current tobacco dependence among the study participants was 18.5%. Amongst people with tobacco dependence, 57.7, 29.5 and 12.8% had moderate, high and very high level of tobacco dependence respectively. All mental health service users with tobacco dependence were males. There was a significant association between tobacco dependence and high school education (AOR 3.02, 95% CI 1.07, 8. 48), alcohol use disorder (AOR 4.14, 95% CI = 1.54, 11.11), daily khat chewing (AOR 13.51, 95% CI = 4.27, 42.74), chewing khat 2–3 times per week (AOR 5.09, 95% CI = 1.37,18.95), chewing khat once a week (AOR 4.31, 95%CI = 1.04,17.78), having friends who smoke tobacco (AOR 4.88, 95% CI = 2.12, 11.25) and being diagnosed with schizophrenia compared to a diagnosis of major depression (AOR 5.26, 95% CI = 2.07, 13.37). However, daily attendance at a place of worship (AOR 0.46, 95% CI = 0.22, 0.95) was associated negatively with tobacco dependence.

**Conclusion:**

In this study, there was a high prevalence of tobacco dependence among mental health services users. There is a pressing need to increase availability of psychological and pharmacological interventions to reduce tobacco dependence and tobacco-related medical illness in this vulnerable group.

## Background

The globalization of tobacco smoking began more than 500 years ago [1]. However, the public health response to the death, disease, and economic disruption that it has caused is less than 50 years old [1]. Nicotine, which is found in tobacco has potential to cause tobacco dependence within a short period of time when people smoke regularly [2]. Tobacco contains 7000 of different chemicals where 69 are carcinogenic [2]. Tobacco smoking causes cancer as well as chronic lung disease such as emphysema, bronchitis, heart disease, and pregnancy related problems [3]. At least, 80% of lung cancer deaths are attributable to smoking tobacco [2]. Tobacco smoking puts a heavy burden on health care systems in developed as well as in developing countries [4]. According to a World Health Organization (WHO) report from 2013, tobacco kills nearly six million people worldwide each year [5]. More than five million of those deaths are the result of direct tobacco use and more than 600,000 are the result of non-smokers being exposed to second-hand smoke [5]. Furthermore, it is predicted that the number of deaths will rise unless current smokers most of who are living in low and middle-income countries stop smoking before middle age [6]. Tobacco smoking among patients with psychiatric disorders is significantly higher than that of the general population [7]. Tobacco smoking has negative effects on physical, mental and the financial well-being of people with mental health problems [8].

In a community based studies from Ethiopia, the prevalence of cigarette smoking varies from 15.4% in Butajira a predominantly area of south central to 28% in Kersa Ethiopia [9–11]. In several studies tobacco dependence has been associated with male gender, tertiary level of education, older age, alcohol use disorders [12], bipolar disorder [12, 13], unipolar depression, personality disorders, schizophrenia and schizoaffective disorder [13, 14]. Although the prevalence of tobacco was studied in different populations in sub-Saharan Africa including Ethiopia, little is known about tobacco dependence and associated factors among mental health service users. Therefore, this study aimed to bridge the knowledge gap by assessing the prevalence of tobacco dependence and associated factors among mental health service users at a teaching Hospital in Southwest Ethiopia.

## Methods

### Study area

A cross-section study was conducted in Jimma University Teaching Hospital (JUTH) which is located in the Southwest part of Ethiopia, 352 km far away from Addis Ababa. JUTH is one of the oldest public hospitals in the country, established in 1937. An out-patient psychiatric clinic was established in 1988 [15] and subsequently a 26 bedded in-patient psychiatric service was developed. Jimma University Teaching Hospital is the only hospital with psychiatry clinic and services in southwest Ethiopia. Data for this study were collected from November 1–30, 2014.

### Data collection instrument

1. Outcome variable: Tobacco dependence

A structured interviewer-administered questionnaire (Fagerstrom Test for Nicotine Dependence (FTND)) was used to assess tobacco dependence. The FTND has six items, with a total score ranging from 0 to 10 to measure nicotine dependence [16]. The FTND has been shown to have good test–retest reliability and validity in populations of smokers with mental health problems [17]. At a cut-off score ≥5, the FTND has good sensitivity and specificity (0.75 and 0.80, respectively) [18]. A total FTND score of five indicates moderate nicotine dependence, a score of 6–7 indicates high nicotine dependence and a score of 8–10 indicates very high nicotine dependence [16]. In this study a total score of FTND ≥5 was considered to indicate tobacco dependence [18].

2. Factors associated with tobacco dependence

*Socio*-*demographic variables*: A structured interviewer-administered questionnaire was used to assess socio-demographic and socio-economic status of participants (age, sex, religion, ethnicity, marital status, educational level, frequency of attending a place of worship, monthly income, occupation and living condition).

*Khat chewing:* A structured interviewer-administered questionnaire was used to assess the pattern of khat chewing, including frequency. In this study, current khat chewing was defined as chewing khat during the month prior to the interview.

*Alcohol use disorders (AUDs):* AUDs was assessed using the four item CAGE questionnaire (Cut down, Annoyed, Guilty, Eye opener). CAGE is short and easily applied in clinical practice. Sensitivity and specificity of CAGE at a cut-off score ≥2 was 0.71 and 0.90, respectively [19]. In this study a total score of ≥2 on CAGE was used to indicate alcohol use disorder.

#### Reasons for starting tobacco smoking

Patients who had tobacco dependence were asked about their reasons for starting tobacco smoking, categorized as follows: smoking for enjoyment, smoking is fashionable, smoking increases self-esteem, self-treatment for their illness, to forget financial problems, to relieve longstanding stress, having friends who use tobacco and family history of tobacco smoking.

#### Clinical characteristics

Charts of the patients were reviewed for psychiatric diagnosis and frequency of hospital visit. A self-reported questionnaire was used to assess co-morbid medical illness, including hypertension, diabetes mellitus and gastritis. The data collection instrument was prepared in English and then translated into both Afan Oromo and Amharic, the main languages spoken in the area. Back-translation into English was undertaken for both languages and the final versions obtained through expert consensus.

### Data collection procedures

Data were collected by five diploma level psychiatric nurses after one day of training to familiarize them with the study tool. Data collection was carried out after the questionnaire has been pre-tested in 5% of the total sample size at JUTH psychiatric clinic. Data collection was supervised by a BSc level psychiatric nurse. The supervisor monitored data quality and checked all questionnaires for completeness.

### Sample size determination and sampling procedure

The sample size was calculated by assuming a prevalence of tobacco dependence of 50%, with the association with any particular outcome to be estimated with a 5% margin of error and 95% confidence interval (∝ = 0.05) and 10% non-response. Based on these assumptions, the sample size for the study was calculated using a single population proportion formula. The total required sample size was 422. All eligible adult patients attending services at outpatient psychiatry clinic during study period were invited consecutively to participate in the study.

### Data analysis

After double data entry verification, data were exported to the Statistical Package for Social Science (SPSS version 20 for windows). Descriptive statistics, including frequencies, percentages, mean and standard deviation was used for describing tobacco dependence. Dependent and independent variables were entered into a bivariate logistic regression one by one in order to identify candidate variables for the final model. All variables associated with tobacco dependence or with a P-value of <0.25 in the bivariate logistic regression were entered together into a multivariable logistic regression by default (enter method) to control potential confounders.

## Results

### Participant characteristics

A total of 422 patients were approached to participate in the study and all agreed to participate (100% response rate). Out of the total study participants, 27.7% (n = 117) and 72.3% (n = 305) of them were females and males respectively. The mean age of study participants was 31.96 (SD 9.89) years. The majority of study participants (71.6%, n = 302) were Oromo ethnicity and followers of Islam religion (62.8%, n = 265). Of the total study participants, 36.3% (n = 153) and 56.4% (n = 238) of them were married and single respectively. Thirty-five percent (n = 101) of the study participants had attended primary school and 34.4% (n = 99) having attended high school. Thirty percent (n = 128) of the study participants had a monthly income of less than 500 Ethiopian Birr (21.25 Euro, 1 Euro = 23.53 Ethiopian Birr), and 28.4% (n = 120) between 501 and 1000 Birr (21.29 to 42.50 Euro), 22.7% (n = 100) between 1001 and 2000 Birr (42.54 and 84.99 Euro) and 17.5% (n = 74) more than 2000 Birr (84.99 Euro) (Table 1).

**Table 1:** Socio-demographic characteristics of mental health service users attending service at Jimma University Teaching Hospital (n = 422)

Characteristics	Frequency (%)
Sex	
Male	305 (72.3)
Female	117 (27.7)
Age (years)	
18–28	181 (42.9)
29–38	156 (37.0)
39–48	47 (11.1)
≥49	38 (9.0)
Ethnicity	
Oromo	302 (71.6)
Amhara	53 (12.6)
Guragie	24 (5.7)
Dawuro	17 (4.0)
Yem	10 (2.4)
Others	16 (3.8)
Marital status	
Married	153 (36.3)
Single	238 (56.4)
Divorced	20 (4.7)
Separated	6 (1.4)
Widowed	5 (1.2)
Religion	
Orthodox	112 (26.5)
Muslim	265 (62.8)
Protestant	41 (9.7)
Catholic	2 (0.5)
Jehovah witness	2 (0.5)
Frequency of attending a place of worship	
Never	39 (9.2)
Some times	226 (53.6)
Daily	157 (37.2)
Education	
Non-literate	74 (17.5)
Read and write only	60 (14.2)
Primary (grades 1–8)	101 (35.1)
Secondary/high school/(grades 9–12)	99 (34.4)
Tertiary (grades more than 12)	88 (30.6)
Occupation	
Employed	71 (16.8)
Unemployed	105 (24.9)
Farmer	84 (19.9)
Merchant	44 (10.4)
Daily-laborer	30 (7.1)
Student	43 (10.2)
House-wife	37 (8.8)
Others	8 (1.9)
Living condition	
Living alone	18 (4.3)
Living with nuclear family	383 (90.8)
Living with relatives	7 (1.7)
Others	14 (3.3)
Frequency of hospital visit	
First visits	32 (7.6)
Two or more visits	390 (92.4)

Other ethnicity = Kefa, Tigray, Siltie and Woliyta

Other occupation = Driver, shoe shine boy, barber and retirement

Other living condition = Living in campus, living in missionary of charity, prison and living with non-relative persons

### Clinical characteristics and other substance use

The most common psychiatric diagnosis was schizophrenia (39.1%, n = 165) followed by major depressive disorder (33.2%, n = 140) and bipolar disorder (16.8%, n = 71). However, other psychiatric disorders were also present, including brief psychotic disorder, schizoaffective disorder, schizophreniform disorder, psychotic disorder not otherwise specified, substance induced psychosis and anxiety disorders accounting in total for 10.9% (n = 46) of participants. More than half (56.6%, n = 239) of the study participants reported chewing khat. Amongst khat chewers, 31% (n = 131) of them were daily user and 86.2% (n = 206) reported that they had chewed khat for more than two years. Nearly one quarter (22.7%, n = 96) of the study participants reported alcohol consumption. Amongst alcohol users, 60.4% (n = 58) fulfilled the CAGE criteria for an alcohol use disorder. Out of the total study participants, 6.4% (n = 27) reported use of other substances including shisha and cannabis.

### Prevalence of tobacco dependence

The prevalence of current tobacco use among study participants was 31.0% (n = 131). Amongst tobacco users, 2.6% (n = 3) and 42.0% (n = 128) were females and males respectively. The most common daily tobacco consumption was between 1 and 10 cigarettes. The prevalence of current tobacco dependence among the study participants was 18.5% (n = 78). All tobacco dependent participants were male. Amongst tobacco dependent participants, 57.7% (n = 45) had a moderate level of tobacco dependence, 29.5% (n = 23) had high levels of dependence and 12.8% (n = 10) had very high levels of tobacco dependence. Among the study participants, 33.6% (n = 142) and 45.7% (n = 193) of them reported a family history of tobacco use and having friends who smoke tobacco respectively. The prevalence of tobacco dependence among participants with alcohol use disorder (AUD) was 51.7% (n = 30). Similarly, the prevalence of tobacco dependence amongst participants who smoked substances like shisha and cannabis was 22.4% (n = 13).

Tobacco dependence was more prevalent among participants who chewed khat daily compared to participants who chewed khat once a week (39.7% vs. 16.7%, P ≤ 0.001). The prevalence of tobacco dependence among patients with bipolar disorder and schizophrenia was 16.9% (n = 12) and 29.1% (n = 48), respectively. However, among participants with major depressive disorder, it was 8.6% (n = 12). The prevalence of tobacco dependence among participants who had a co-morbid medical illness was 17.9% (n = 7).

### Reasons reported for initiating tobacco smoking

Nearly 19.4% (n = 82) of the study participants reported that they had started tobacco smoking to feel pleasure and 10% (n = 42) of them thought tobacco smoking is fashionable. However, 2.4% (n = 10) and 7.6% (n = 32) of them reported that they started tobacco smoking as self-treatment for their illness and to increase self-esteem, respectively. Amongst khat chewers, 8.7% of them reported that they started tobacco smoking as self-treatment for their mental illness (see Table 2).

**Table 2:** Reasons for initiating tobacco smoking among mental health service users at Jimma University Teaching Hospital, Southwest Ethiopia

Reasons of tobacco smoking	Frequency (%)
Enjoyment	82 (19.4)
Smoking is fashionable	42 (10)
Increase self-esteem	32 (7.6)
Self-treatment for their illness	10 (2.4)
To relief longstanding stress	6 (1.4)
To forget financial problems	1 (0.2)
Family history of tobacco smoking	142 (33.6)
Having friends who use tobacco	193 (45.7)

### Factors associated with tobacco dependence

In the multivariable logistic regression model (see Table 3), having attended high school education (AOR 3.02, 95% CI = 1.07, 8. 48), being diagnosed with schizophrenia (AOR 5.26, 95% CI = 2.07, 13.37), alcohol use disorder (AOR 4.14, 95% CI = 1.54, 11.11), daily khat chewing (AOR 13.51, 95% CI = 4.27, 42.74), chewing khat 2–3 times per week (AOR 5.09, 95% CI = 1.37, 18.95), chewing khat once a week (AOR 4.31, 95% CI = 1.04, 17.78) and having friends who smoke tobacco (AOR 4.88, 95% CI = 2.12, 11.25) were associated positively with tobacco dependence. However, daily attending a place of worship (adjusted Odds Ratio (AOR) 0.46, 95% CI = 0.22, 0.95) was negatively associated with tobacco dependence (see Table 3).

**Table 3:** Multivariable logistic regression of factors associated independently with tobacco dependence among mental health service users at Jimma University Teaching Hospital, Southwest Ethiopia (n = 422)

Variables	P-value	AOR	95% CI
Frequency of attending a place of worship			
Never		1	
Sometimes	0.08	0.32	0.09, 1.12
Daily	0.04*	0.46	0.22, 0.95
Education attended			
No formal education		1	
Primary	0.191	1.94	0.71, 5.24
Secondary	0.036*	3.02	1.07, 8.48
Tertiary	0.089	2.95	0.84, 10.31
Psychiatry diagnosis			
Major depression		1	
Schizophrenia	<0.001**	5.26	2.07, 13.37
Bipolar disorder	0.072	2.76	0.91, 8.38
Other psychiatric disorders	0.851	1.14	0.27, 4.69
Alcohol use pattern			
Abstinent		1	
Alcohol use but not disorder	0.951	0.96	0.29, 3.19
Alcohol use disorder	0.005*	4.14	1.54, 11.11
Khat chewing			
Never chewing		1	
Daily chewing	<0.001**	13.51	4.27, 42.74
2–3 times per week	0.015*	5.09	1.37, 18.95
Once a week	0.043*	4.31	1.04, 17.78
Having friends who smoke tobacco			
Yes	<0.001**	4.88	2.12, 11.25
No		1	

Other psychiatric disorders = brief psychotic disorder, schizoaffective, schizophreniform, psychotic disorder not otherwise specified, substance induced psychosis and anxiety disorders

* P < 0.05; ** P < 0.001

## Discussion

In this cross-sectional survey of mental health service users at a hospital in Southwest Ethiopia, nearly one-fifth of them had probable tobacco dependence (18.5%). The overall prevalence of current tobacco dependence found in this study (18.5%) was lower than similar studies carried out in Iran (28.75%) [20] and in Brazil (59.0%) [21]. This may be due to the difference between screening tools in Brazil and our study (DSM IV vs. FTND). Socio-cultural differences between Ethiopia, Brazil and Iran may also have contributed to the difference. The prevalence of current tobacco smoking found in this study (31%) was also lower than that found in other studies, for example in India (36%) [12]. However, the prevalence of current tobacco smoking found in this study (31%) was consistent with the community based study from Kersa, Ethiopia, (28%) [10].

The prevalence of moderate (57.7%) levels of tobacco dependence found in this study was higher than a similar study done in Brazil (13.5%) [14]. However, the prevalence of high levels of tobacco dependence found in our study (29.5%) was consistent with study done in Brazil (29.2%) [14]. The prevalence of high levels of tobacco dependence found in our study (29.5%) was lower than a similar study conducted in Iran (64.4%) [22]. The difference may be due to the difference in cut-off point. The Iranian study used a cut-off point of >3 FTND score. However, in this study we used a 6–7 FTND score for high level of tobacco depedence. The prevalence of tobacco dependence among patients with schizophrenia and bipolar disorders found in this study (29.7% and 16.9%, respectively) was consistent with a similar study done in Karman Iran (30.9 and 22.9%, respectively) [20]. In our study, all tobacco dependent participants were males which are in keeping with the pattern of tobacco use in the general population in Ethiopia and reflect cultural influences on females not to smoke tobacco.

Daily attendance at a place of worship was negatively associated with tobacco dependence compared to never attending at a place of worship. This may be due to patients who were attending a place of worship more frequently might be more likely to receive religious advice not to smoke tobacco. Daily khat chewing was associated with tobacco dependence. This may be due to patients and their caregivers also explained their ongoing khat use was necessary to improve the patient’s functioning and to alleviate medication side effects [23]. In our study being diagnosed as schizophrenia was associated with tobacco dependence, consistent with the Brazil study [14]. This may be because nicotine intake appears to improve some cognitive impairments and medication side effects among patients with schizophrenia [24]. In this study, having friends who smoked tobacco was associated with tobacco dependence. This may be due to presence of peer pressure to use tobacco and other substances. However, the mechanisms by which people with mental illness start to smoke cigarettes need further investigation.

Attending high school education was found to be associated with tobacco dependence, in keeping with the India study [12]. Presence of an alcohol use disorder was also associated with tobacco dependence in the India study [12]. Patients who chewed khat daily were more often identified to have tobacco dependence than patients who chewed khat once a week (39.7% vs. 16.7%, P ≤ 0.001). This may be due to the fact that patients use different substance to get relief from side effects of antipsychotic medications. Also, patients with schizophrenia may use different stimulants in order to get rid of their negative symptoms.

A limitation of our study is that the Fagerstrom test nicotine dependence (FTND) had not been validated for use in Ethiopia. Furthermore, FTND is a screening tool and may not give an accurate estimation of tobacco dependence. Social desirability bias could be another limitation as patients with tobacco dependence might minimize or deny their tobacco use. We have used a cross-sectional study design which does not allow investigation of cause and effect relationships. Also our sampling method was another limitation for the representatives of the results to all people with mental illness in Ethiopia.

The hospital in which the study was undertaken is the only hospital with psychiatry clinic and services in southwest Ethiopia. Therefore, selection of a single institution to represent people with mental illness in Southwest Ethiopia is due to absence of other center that provides psychiatry service in the area and patients who agreed to participate in the study were consecutively invited to participate in the study. There is evidence about the appropriateness of consecutive sampling in a clinical setting when a disease varies by season. Simple random sampling would have been the optimal approach. Further, community based research with a robust random sample is needed to determine the prevalence of tobacco dependence among mentally ill peoples residing in Southwest Ethiopia.

## Conclusions

A high prevalence of tobacco dependence was found among people with schizophrenia attending mental health services in Ethiopia. Psychotherapy and pharmacotherapy are crucial interventions to reduce tobacco dependence and tobacco related medical illness in this population. Patients with tobacco dependence also tended to use other substances like khat and alcohol which have their independent impact on medication adherence and prognosis of the mental illness. A comprehensive treatment approach is necessary to reduce the risk associated with these substances.
